# Clinical analysis and misdiagnosis of cerebral venous thrombosis

**DOI:** 10.3892/etm.2012.697

**Published:** 2012-09-04

**Authors:** XIAOTONG WANG, XUWEN SUN, HONG LIU

**Affiliations:** Department of Neurology, Yantai Yuhuangding Hospital, Yantai 264000, P.R. China

**Keywords:** cerebral venous thrombosis, headache, intracranial hypertension, misdiagnosis

## Abstract

The present study aimed to summarize the clinical characteristics and experiences of misdiagnoses of cerebral venous thrombosis (CVT). A total of 18 patients with CVT who received treatment between September 2009 and August 2011 were enrolled. Of the patients, 13 were females and 5 were males with an average age of 39 years. Clinical manifestations and characteristics according to cerebrospinal fluid examination and imaging were summarized retrospectively. CVT principally manifested as headaches, papilledema, psychiatric symptoms, impaired consciousness and seizure disorders, with or without neurological defects. A combination of magnetic resonance imaging (MRI) and venography (MRV) was demonstrated to be an effective method for CVT diagnosis. Of the 18 patients, 8 were misdiagnosed, as a result of the lack of further examination due to undefined etiology, atypical clinical manifestations and ambiguous neuroimaging direct signs. The clinical symptoms of CVT are aspecific, as a result of which misdiagnosis tends to occur. For patients with intracranial hypertension accompanied with or without neurological defects, MRI combined with MRV can improve the accuracy of CVT diagnosis.

## Introduction

Cerebral venous thrombosis (CVT) is a rare type of cerebrovascular disease, which accounts for approximately 0.5–2% of all stroke cases (1,2). CVT has varied and atypical clinical manifestations, as a result of which misdiagnosis and missed diagnosis often occur (3). The majority of CVT cases are accompanied by intracranial hypertension (4). CVT principally manifested as headaches, focal neurological deficits, seizure disorders and impaired consciousness; other symptoms include papilledema, abnormal vision, nausea, emesis, cranial nerve lesion and coma (5). These varied and atypical clinical manifestations often pose difficulty in early diagnosis of CVT, as a consequence of which necessary treatment is delayed. Early treatment can greatly improve the prognosis of CVT, save patients’ lives, improve their life quality, and reduce family and social burdens. In recent years, with the development and wide adoption of neuroimaging, the diagnosis rate of CVT has markedly increased. Some CVT patients can be diagnosed using computed tomography (CT), most can be confirmed by magnetic resonance imaging (MRI) combined with magnetic resonance venography (MRV), and only a small number require diagosis by further digital subtraction angiography (DSA).

The current study was based on case analyses of 18 CVT patients who received treatment between September 2009 and August 2011, as well as the experiences of their treatments.

## Patients and methods

### General data

A total of 18 CVT patients including 5 males and 13 females were involved. Their ages ranged from 22 to 65 years with a meane age of 39. The time from disease onset to admission to hospital ranged from 1 day to 45 days with an average of 7 days. The causes included late trimester of pregnancy (one case), puerperium (four cases), upper respiratory tract infection (three cases), brain trauma (one case), abnormal immune system (two cases), oral administration of contraceptives (two cases), and uncertain causes (five cases).

The current study was conducted in accordance with the Declaration of Helsinki and approved by the Ethics Committee of Yantai Yuhuangding Hospital. Written informed consent was obtained from each participant.

### Clinical manifestations

Headaches were found in 15 patients, which was the primary symptom among the recruited participants. Other symptoms included papilledema in 12 patients, abnormal eye movements and diplopia in one, emotional disturbance in one, unconsciousness in five, seizure disorders in six, language disorders in two, limb paralysis in six, and positive meningeal irritation signs in six.

### Auxiliary examinations

CT was performed for 15 patients: five patients were normal; one was suspected of an ‘Δ’, three were observed with lobar hemorrhage, one with embolic infarction in the left parietal lobe, one with hypodensity in the right frontal and parietal lobes, one with a large area of mixed densities in the left frontal lobe, two with hypodensity in the bilateral thalamic basal nuclei, and one with subarachnoid hemorrhage. MRI and MRV were performed for 16 patients. No abnormality was observed in one patient, superior sagittal sinus thrombosis was observed in seven, inferior longitudinal and straight sinus thrombosis in three, bilateral transverse and sigmoid sinus thrombosis in two, and great cerebral venous thrombosis in three. MRI alone was performed for one patient: the patient presented with a large area of bleeding in the left temporal lobe, a median line malposition, a brain herniation, and absence of the flow void signals in the right jugular vein and the left transverse and sigmoid sinuses (enhancement scanning did not show enhanced images in these veins). One patient who was observed as normal according to MRI + MRV also received DSA and the result disclosed partial visualization of the cerebral venous and venous sinuses and cerebral circulation time prolonging by 13 sec [less than 9 sec is the normal value, and longer circulation time indicates a poorer prognosis (6)]. DSA for the patient with subarachnoid hemorrhage confirmed bilateral transverse and sigmoid sinus thrombosis. Lumbar puncture for cerebrospinal fluid (CSF) examination was performed for 14 patients: three had CSF pressures between 200 and 250 mmH_2_O (1 mmH_2_O = 0.098 kPa), six had CSF pressures between 250 and 350 mmH_2_O, and five had CSF pressures of >350 mmH_2_O, one of whom had uniform bloody CSF; eight had basically normal CSF cell counts as well as normal protein concentrations; four had protein concentrations between 0.9 and 1.2 g/l; and two had abnormal white blood cell counts (15×10^6^/l and 34×10^6^/l), which were mainly represented by monocytes, and protein concentrations of 0.68 g/l and 0.87g/l but with normal glucose and chloride concentrations.

### Treatment and turnover

After final diagnosis, each patient was given a combined modality therapy such as anticoagulation, thrombolysis, platelet inhibition, intracranial pressure reduction through cerebral edema remission, and complication prevention, depending on his/her condition. Patients with seizure disorders were administered anti-epileptic treatment and those with concurrent infections were administered anti-infective therapy. A total of 14 patients exhibited stable pathogenetic conditions and were discharged from the hospital, with eight completely recovered, two with varying degrees of carryover limb paralysis (one still with language disorders), one with diplopia, two with slow response, and one in a vegetative state. After the discharge, 11 patients were on oral warfarin based on a moderate international normalized ratio (INR) between 2.0 and 3.0. One patient was administered bayaspirin (Bayer Health Care Company Ltd, Germany) as a replacement for warfarin for platelet inhibition due to inaccessible prothrombin time monitoring. Among the 18 patients, 2 succumbed to their condition during hospital stay, one was transferred to a higher level hospital due to a serious condition but succumbed to their condition the next day, and one discontinued the treatment and left the hospital.

## Results

### Clinical characteristics

The CVT cases in the present study were analyzed and the characteristics were summarized as follows: i) CVT tends to occur among young adults, has an average onset age of 39 years, and females are more likely to suffer from this disease than males with a 1:2.5 gender ratio; ii) the majority of CVT cases exhibit acute onset and progressive aggravation; iii) CVT is principally manifested by headaches, emesis, papilledema, and sometimes blurred vision; iv) neurological impairments such as limb paralysis and language disorders may or may not be present; v) seizure disorders or emotional disturbance may occur in company; and vi) the majority of CVT cases occur during pregnancy and puerperium, but some are caused by unknown reasons.

### Misdiagnoses

**Figure 1:**
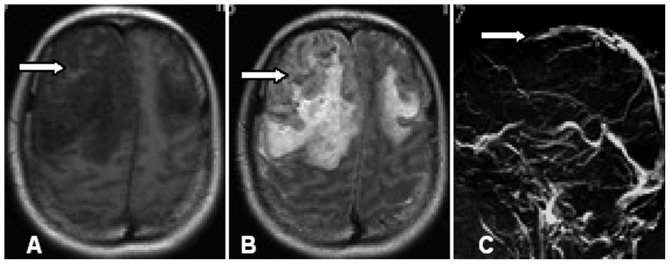
Images of CVT affecting bilateral superior sagittal sinuses by MRI + MRV. (A and B) Long T1 and T2 signals in the bilateral frontal lobes and spot-like slice equal T1 and T2 signals in the focus according to MRI (indicated by the arrows); (C) absence of the blood flow signal at the anterior segment of the superior longitudinal sinus according to MRV (indicated by the arrow).

There were eight misdiagnosed patients in this study: one was misdiagnosed with cerebral hemorrhage, one with hemorrhagic cerebral infarction, one with cerebral infarction, one with subarachnoid bleeding, one with increased intracranial pressure, two with viral encephalitis, and one was misdiagnosed with intracranial space-occupying lesion and received neurosurgical treatment, but was later transferred to the Department of Neurology after the disclosure of superior sagittal sinus thrombosis by MER + MRV. The misdiagnosed cases are reported briefly as follows:

*Case 1*.A patient was hospitalized for more than 20 days of headaches. Medical examination revealed that papilledema was more popular than other neurological focal signs. CT and MRI + MRV were performed successively, but no abnormalities were observed. Lumbar puncture disclosed a CSF pressure of 280 mmH_2_O, and thus he was preliminarily diagnosed with increased intracranial pressure. After the doctors’ consultation, DSA was performed. The results revealed that the patient had partial visualization of the great cerebral vein and prolonged cerebral circulation time.
*Cases 2 and 3*. CVT in two patients initially manifested as headaches and fevers, with one accompanied by mental symptoms and the other by meningeal irritation signs. Lumbar puncture disclosed that they had increased intracranial CSF pressures and mildly increased cell counts and CSF protein concentrations. MRI did not display any abnormality in one patient but revealed hypointensity on the T1-weighted image (WI) and hyperintensity with small foci at the midst of the T2 WI of the left temporal lobe in the other. The patients were diagnosed preliminarily with viral meningitis. After hospitalization, they received antiviral and dehydration treatments, but their conditions were aggravated. One patient was subjected to lumbar puncture. The results showed that her CSF pressure increased, albeit without noticeable routine or biochemical changes. Cerebral MRV was subsequently performed, and the results showed superior sagittal sinus thrombosis. The patient received active of anticoagulation and thrombolysis treatments, and was cured one month later. The pathogenetic condition of the other patient deteriorated abruptly and symptoms of consciousness disorders, convulsion, and anisocoria appeared. Emergency MRI was performed for her, and the result showed she had a large area of bleeding in the left temporal lobe, a median line malposition, a brain herniation, and absence of the flow void signals in the right jugular vein left and the transverse and sigmoid sinuses (enhancement scanning further confirmed this result). All rescue measures proved ineffectual and the patient succumbed to her condition.
*Case 4*. A patient was hospitalized for left limb weakness. Cerebral CT showed hypointensity in her right frontal parietal lobe. The patient was first diagnosed with cerebral infarction. However, the patient later presented with aggravated symptoms with apparent headaches and projectile vomiting. MRI + MRV exhibited superior sagittal sinus thrombosis and bilateral parietal venous infarctions accompanied by hemorrhage.
*Case 5*. A male patient was hospitalized after three days of a headache and one day of right limb weakness. CT showed cerebral hemorrhage in his left fronto-parietal junctional region. The patient was treated according to the symptoms of cerebral hemorrhage. However, the curative effect was poor: his headache was aggravated and consciousness disorders manifested. Further MRI + MRV revealed superior sagittal sinus thrombosis, an expanded fronto-parietal bleeding size, and a median line malposition.
*Case 6*. A patient presented with a headache and left-side paralysis. CT showed that the patient had a right parietal hemorrhagic infarction. Following treatment, the patient’s condition deteriorated. Further MRI + MRV disclosed superior sagittal sinus thrombosis and bilateral fronto-parietal venous infarctions accompanied by hemorrhage, with the right side more serious (Fig. 1).
*Case 7*. A patient suffered from headaches and emeses. Medical checkup showed positive meningeal irritation signs in the patient. Lumbar puncture and cerebral CT were performed. The results showed that the patient had uniform bloody CSF with a pressure of 310 mmH_2_O and subarachnoid bleeding. Two days later, DSA disclosed bilateral transverse and sigmoid sinus thromboses in the patient.
*Case 8*. The patient who was transferred from the Department of Neurosurgery to the Department of Neurology received cerebral CT for headaches and one-sided limb weakness. The result showed a large area of mixed densities in her left frontal lobe. The patient was misdiagnosed with intracranial space-occupying lesion.

## Discussion

CVT is a rare type of ischemic stroke. Its clinical manifestations are varied and atypical due to lack of fixed diseased regions, varying degrees of lesion severity, and different progression velocities; these characteristics render definite diagnosis of CVT difficult (7).

CVT can be induced by a variety of factors, such as severe dehydration, external injuries, hematological diseases, heart diseases, pregnancy, infections, immune abnormalities and oral contraceptives; in addition, 20% of CVT cases are due to unknown causes (8). Consequently, in patients with CVT of unknown causes, the rates of misdiagnosis and delayed diagnosis are also higher. In the present study, 4 of the 8 misdiagnosed cases were due to uncertain causes.

Females are more likely to suffer from CVT, accounting for 70–80% of all CVT patients (9). In this study, 13 of the 18 CVT patients were females. Furthermore, among the 13 cases, one occurred during gestational period and four occurred during puerperium. Women in the perinatal period are the high-risk population for CVT, as the concentrations of multiple blood coagulation factors and fibrinogen increase and the blood stays in a hypercoagulable state in this period, together with various factors at the time of delivery such as a blood loss, greater perspiration, hypovolemia, increased blood viscosity, and a decelerated speed of blood flow. Bearing this in mind, in most circumstances, clinicians are alert for the occurrence of CVT whenever patients in perinatal period show symptoms of headaches and emeses with or without focal neurological deficits. Therefore, misdiagnosis of CVT in this population is rare.

Headache is the most common symptom of CVT, or even the only symptom in some cases (10), with an incidence of 75–95% in patients with CVT (6). The site of headache bears no direct correlation with the site of sinus thrombosis, except that approximately 61% of patients with sigmoid sinus thrombus alone or with transverse sinus thrombus have pains in the occipital and neck regions (7,11). In addition, headaches may have varying degrees of severity in CVT patients. Generally, a severe headache attracts attention more easily from the patient and clinician. However, when headache represents the only symptom without other neurological symptoms or signs, misdiagnosis tends to occur, as in the first case in this study, which was misdiagnosed for increased intracranial pressure. For patients with symptoms of headache and upper respiratory tract infection, misdiagnosis as viral meningitis frequently occurs (there were two cases of such misdiagnosis in this study). Furthermore, in addition to the common symptoms such as headache and papilledema, certain CVT patients may present with focal neurological impairments such as limb paralysis and language disorders, or with seizure disorders and emotional disturbance. For these patients, especially those whose imaging results show cerebral infarction, cerebral hemorrhage, or intracranial space occupation, misdiagnosis as a certain disease according to the imaging result is also likely to occur. In this study, this factor was responsible for the majority of the misdiagnosed cases.

The direct signs of cerebral CT reflect empty triangle and cord signs, thereby providing important clues for early diagnosis of CVT. However, although these signs have high specificity, they have the drawback of a low positive rate. In this study, only 1 out of 18 patients presented with an atypical empty triangle sign. The indirect signs of cerebral CT can disclose focal oedema or infarction and hemorrhage.

MRI combined with MRV is currently the preferred atraumatic and effective diagnostic method for CVT (12,13). MRI combined with MRV has even been considered as the gold standard for diagnosis of CVT by certain investigators (14). In this study, more than 90% of the patients were observed with abnormalities using this method.

The imaging results showed that most lesions affecting the superior sagittal sinus were located in the frontal and parietal lobes, and the affection was either unilateral or bilateral (Fig. 1); lesions affecting the transverse and sigmoid sinuses were primarily located in the temporal lobe or in the tempo-occipital junctional region and were manifested by localized edema, infarction (ischemic or hemorrhagic, which does not comply with the distribution law of arterial blood vessels), intracranial or cerebral ventricular hemorrhage, and subarachnoid hemorrhage. Furthermore, this study showed that disappearance of the flow void signals in venous sinuses and changes in signal intensity are both capable of indicating venous thrombosis. MRV can display not only the absence, constriction, edge unsharpness, and filling defects of the blood flow signals in the affected venous sinuses, but also the conditions of the relevant collateral pathways. Being a noninvasive blood vessel examination method, MRI + MRV is considered to be an effective measure for CVT diagnosis. However, MRI + MRV has a limitation, namely that anatomical variations and defective development of a venous sinus often lead to the appearance of false positivity. DSA can effectively overcome such a limitation and therefore has been widely accepted as the gold standard for CVT diagnosis. This method can clearly demonstrate a thrombosed site as well as the scope of the thrombus and dynamically monitor venous abnormal back flow and compensatory circulation. However, as DSA is invasive, it has not been applied in routine examination to date.

CSF examination through lumbar puncture can also provide important information for CVT diagnosis. CVT patients have increased intracranial CSF pressure, which is an important sign. In addition, the majority of patients have normal or slightly increased protein concentrations and cell counts. For patients in whom these indices significantly increase, due attention should be given to the possible occurrence of other diseases or CVT complicated by other diseases, such as infections and tumors.

Currently, anticoagulation therapy is the primary form of CVT treatment, which is also applicable to CVT patients accompanied by intracranial hemorrhage. Specifically, anticoagulant therapy can take the following forms: heparin is administered subcutaneously or intravenously to double the activated partial thromboplastin time; or warfarin is administered orally and the INR of the treatment is adjusted to 2.0–3.0 (the recommended application duration is three months to half a year, which can be adjusted according to patients’ conditions). Other medical treatments include dehydration to reduce intracranial pressure, and active treatments of seizure activity and emotional disturbance. Furthermore, the causes of CVT, such as immunological and hemorheological abnormalities, should also be targeted in treatment.

Endovascular interventional treatment may also employ local thrombolysis. Drugs that can be used in this type of therapy include urokinase and recombinant tissue type plasminogen activator. Many investigators hold the view that local thrombolysis therapy can achieve a greater curative effect than anticoagulant therapy (15,16), on severe and progressive CVT in particular (17,18). Nevertheless, other therapies, such as balloon or catheter embolectomy and endovascular stent plasty can be used for CVT treatment. However, as these procedures are difficult to operate, costly and invasive, they have not been widely applied in clinical practice.

For patients with severe CVT, especially severe CVT with signs of cerebral hernia, decompressive craniectomy is an effective way to increase survival rates (19).

**Figure 2:**
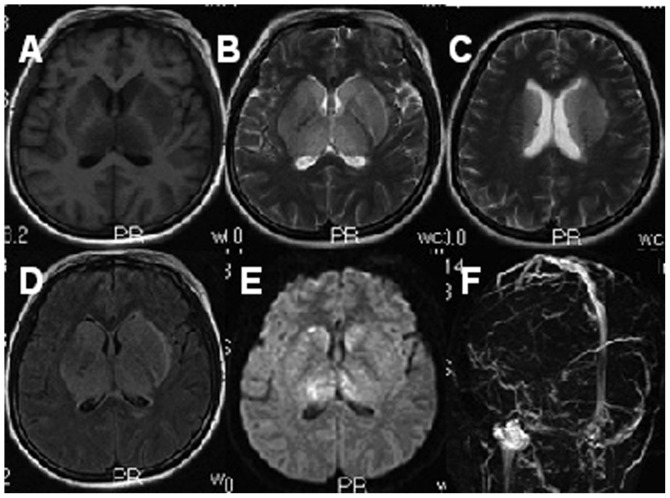
(A–C) Long T1 and T2 signals with hyperdense FLAIR signals in the bilateral thalamus and basal ganglia regions according to MRI, as indicated by the arrows; (E and F) non-seriously restricted diffusion according to diffusion-weighted imaging; (F) inferior sagittal and straight sinus thrombosis.

CVT is a relatively rare type of ischemic cerebrovascular disease; in recent years, the mortality rate of CVT has been reduced to 6–30% due to markedly improved diagnosis and recovery rates (20). These improvements appear to be directly creditable to the technical development of medical imaging. However, dependence on medical imaging alone may cause misdiagnosis for other diseases such as cerebral infarction, cerebral hemorrhage, subarachnoid hemorrhage and tumor. Therefore, based on the experience in the treatment of CVT, to further increase the diagnosis and recovery rates, the occurrence of CVT cannot be excluded in the following conditions: when i) patients exhibit headache, fever, and increased CSF protein concentration and cell count, and these symptoms cannot be improved or are even aggravated following anti-infection treatment; ii) cerebral hemorrhage does not fit with the distribution law of arterial blood vessels, particularly in patients with non-seriously restricted diffusion according to diffusion-weighted imaging (vasogenic edema; Fig. 2), or cerebral hemorrhage does fit with the characteristics of hypertensive cerebral hemorrhage but these lesions fail to explain the manifestations of intracranial hypertension such as headache and fundus edema; iii) patients are observed with infarction foci in the bilateral thalamus and basal ganglia regions according to CT or MRI (high attention should be given to the possible occurrence of great cerebral venous and straight sinus thromboses; Fig. 2); iv) patients are observed with benign intracranial hypertension according to clinical examination and with atypical intracranial space-occupying lesion according to imaging; and v) patients, particularly adult patients, exhibit headache and focal neurological deficits of uncertain causes, and examination cannot completely explain the symptoms. Furthermore, CT and MRI + MRV are simple and feasible methods in CVT diagnosis, and further DSA can be performed when necessary.
